# Terminology and descriptions of navigation and related practices for children with neurodisability and their families: a scoping review

**DOI:** 10.1186/s12913-022-07617-y

**Published:** 2022-02-17

**Authors:** Emily Gardiner, Vivian Wong, Grace Lin, Anton R. Miller

**Affiliations:** 1 BC Children’s Hospital Research Institute, 950 West 28th Avenue, Vancouver, BC V5Z 4H4 Canada; 2 Division of Developmental Pediatrics, Department of Pediatrics, University of British Columbia, 4480 Oak Street, Vancouver, BC V6H 3V4 Canada; 3 Sunny Hill Health Centre at BC Children’s Hospital, 4500 Oak Street, Vancouver, BC V6H 3N1 Canada; 4 School of Medicine, Queen’s University, 15 Arch Street, Kingston, ON K7L 3N6 Canada

**Keywords:** Neurodisability, Patient Navigation, Navigation, Scoping Literature Review, Coordination, Family Support, Key Working, Coaching

## Abstract

**Background:**

Children with neurodisability (ND) represent a significant population with a demonstrated need for coordinated support. Patient navigation has a primary focus on: facilitating access to and connection amongst fragmented systems; as well as the provision of educational and emotional support. Given the distinct needs of children with ND and their families, programs built upon such core concepts could be of great benefit. The diversity of terminology encompassing navigation-related concepts and activities (e.g., care coordination, case management, family support), however, presents challenges to both practice and research. This scoping review examined the terminology and descriptions provided within published articles on navigation-type models for children with ND and their families.

**Methods:**

The scoping review was conducted according to the Joanna Briggs Institute methodology. A preliminary search was completed on PubMed (NCBI), MEDLINE (Ovid) and CINAHL (EBSCO) to identify initial search terms, upon which a full search strategy was developed and executed in MEDLINE (Ovid) and CINAHL (EBSCO). After screening records according to our inclusion and exclusion criteria, a full-text review of relevant articles was conducted and data extracted using a researcher-developed tool. Under close supervision by the research team, study selection was primarily performed by one author.

**Results:**

Of the 2597 papers identified, 33 were included in the final review. From the included papers, a total of 49 terms were extracted, 20 of which were unique. Across the diversity of terminology observed, articles provided detailed and rich descriptions characterized by four central domains, namely: (*i*) what navigation-related resources, supports and services aim to *facilitate* and *(ii) provide; (iii)* descriptions of their *intended outcomes;* as well as (iv) *guiding principles*.

**Conclusions:**

This scoping review addresses a gap in our knowledge related to the specification of patient navigation and related supports as applied to the specific context of children with ND and their families. Given the particular needs of this population, we propose an empirically-informed integrative model that synthesizes the findings from this scoping review. We suggest that this framework can be used as a guide to the mindful characterization of how supports aiming to connect children and families to needed service are termed and described within future research and in practice.

**Supplementary Information:**

The online version contains supplementary material available at 10.1186/s12913-022-07617-y.

## Background

Children with neurodisability (ND) represent a diverse and significant^1^ cohort of the pediatric population with considerable and unique support needs. According to Morris et al. [3], “neurodisability” refers to:

a group of congenital or acquired long-term conditions that are attributed to impairment of the brain and/or neuromuscular system and create functional limitations. A specific diagnosis may not be identified. Conditions may vary over time, occur alone or in combination, and include a broad range of severity and complexity. The impact may include difficulties with movement, cognition, hearing and vision, communication, emotion, and behaviour. (p. 3-4)

The reality that children’s needs fall across multiple domains of functioning and manifest in myriad ways means that supports have to be accessed from various, and oftentimes distinct sources, with the burden for coordination being left to the family. For example, needed supports may be accessed through both public and private sources, cross the sectors of health, education, and social service, and may be received from primary, secondary, or tertiary levels of care. Moreover, available financial support varies considerably by region and also according to the child’s diagnosis, resulting in scenarios in which some conditions are the “haves” and others the “have-nots”, compounding disparities in the ease with which families access support [4, 5].

Indeed, the many service-related barriers experienced by children with ND and their families are well documented [6]. Caregivers encounter significant challenges with accessing information and services, experience a lack of communication and coordination within and across agencies, and perceive the responsibility to coordinate their child’s services as overwhelming [7–9]. These experiences negatively impact family quality of life [10] and family burden [11], and those of low socioeconomic status [12, 13] or who are immigrants [14, 15] are further at risk.

Given all these barriers and challenges, and their impact on family life, it is clear that children with ND and their families are in need of concerted efforts that not only facilitate their access to needed services, but that can effectively coordinate them. One such well-suited initiative may be patient navigation (PN), a model that emerged in the early 1990s through the work of Dr. Harold Freeman, who observed significant improvements in early detection and survival among breast cancer patients paired with a PN program [16]. Although defined and described in various ways, PN assists ‘patients’ and families by facilitating access to and coordination amongst services, as well as with the provision of educational and emotional support [17]. Other core roles associated with PN include advocating for the family and for broader system change, as well as facilitating identification and reduction of barriers, peer connections, and transition planning [18]. The core aims of PN directly align with the service-related challenges identified by families of children with ND, and have great potential to positively impact the health outcomes of both these children and their families in the short- and long-term [19]. Emerging research supports the efficacy of this approach for children with ND and their families, and is associated with improved access to and knowledge about services, as well as reduced parental stress [20–23] (see [18] for a more in-depth review of this emerging research).

Descriptions of caregivers’ challenges with accessing and coordinating services for their children with ND are not a new phenomenon in the literature (e.g., [6, 24]). Indeed, Agosta and Melda noted in 1995 that interagency collaboration was a key component of ‘family support’ that was critically lacking [25]. However, the application of PN, specifically, at least within the context of children with ND, is relatively new and rare. This suggests that other terms are being used to describe *practices* centred on connecting children and families to supports and coordination amongst services. For example, Majnemer et al. [26] describe a “coaching” model for young children awaiting developmental assessment that includes many of the same elements (e.g., educational support, peer networking, access to resources). Although not specific to children with ND, Carter et al. [27] found that across 34 papers describing navigation in primary care settings, navigators were identified by 15 unique titles, such as “Patient Navigator”, “Case Manager”, “Healthy Living Coach”, and “Program Coordinator”. The presence of these varied terms, which all appear to encompass and overlap with PN-related or aligned concepts and activities, challenges the field at both practical and academic levels [28]. This diversity of titles is confusing for service planners and policy makers, who may lack clarity regarding how to “package” and present initiatives aimed at assisting families to navigate service systems. Families are also challenged, as they are unlikely to know what subtle role differences may be associated with each term, and therefore which service provider would best meet their needs. Similarly for researchers, the lack of consistency makes it difficult to evaluate and compare models that have aligned components and aims, but which operate under different service umbrellas (i.e., “case management” versus “navigation”).

The current scoping review aims to unpack and provide a degree of clarity to the range of terminology and descriptions that have been provided in the literature pertaining to the practice of connecting children with ND and their families to supports and services in their communities. This represents an important first step in addressing gaps in the literature. A notable gap is that children with ND are poorly represented in the literature. As children with ND have not been identified as the population of interest within any of the recently published scoping reviews on PN [27, 29–32], it is critical that the unique and complex service-related needs of this group are considered. A second major deficiency is that existing literature pertaining to “navigation” versus “case management” versus “care coordination”, for example, exists in siloes, which brings the risk of each program or field reinventing the wheel, neglecting to synthesize and integrate pearls of wisdom from across the various streams, and missing opportunities to appropriately tailor interventions to the population’s particular needs [30]. In order to understand and ultimately improve the experience and outcomes of families of children with ND interacting with fragmented service systems, the terminology and concept of PN relative to this particular experience and context must first be clarified and understood. The current scoping review examined the following question:

What *terminology*, and what *descriptions* have been provided in the literature pertaining to navigation-type resources, supports, and activities aimed at connecting children with ND and their families to community-based supports and services?

## Methods

### Search strategy

The search strategy adhered to the JBI methodology for scoping reviews [33], and was developed in collaboration with an information specialist/clinical librarian (see Lin et al. [34] for the complete protocol for the current study, including the full search strategy). Relevant electronic databases including Medline (OVID) and CINAHL (EBSCO) were searched in June 2019 for literature published between 1990 and 2019 pertaining to navigation and navigation-type resources, supports, and activities. The year 1990 was chosen as this was when PN first emerged as a concept [16]. The reference lists of included articles were also screened for additional relevant studies. The decision to include varied terminology in our search strategy, as opposed to limiting it to the term “navigation”, was made following discussion in the literature of the overlapping nature of various terms [28], as well as based on discussion with community partners representing family advocates, community-based professionals, and policy makers who communicated that the use of such terminology in varied and confusing ways was a barrier to the advancement of the field. This review was undertaken as part of a larger research project guided by an advisory group, whose members are involved in the practice and policy of service delivery for children with ND and their families, with principal focus on supporting families on their ‘journey’ to accessing needed supports. We therefore felt that our approach would be most reflective of this, at times messy reality, and had the greatest potential to move the field toward development of an inclusive terminology.

## Inclusion criteria and study selection

The current review considered papers according to specified inclusion and exclusion criteria related to participants, concept, context, and types of sources (see Table 1). ProQuest RefWorks was used throughout to manage article retrieval and screening, and study selection followed five steps. First, all retrieved citations resulting from the utilized search strategy were uploaded to RefWorks software, and duplicates were removed. Second, all titles and abstracts were screened against the inclusion criteria. If it was unclear as to whether a paper met these criteria based on their title and abstract, it was included in the next stage of review. Third, full texts of relevant papers were retrieved and the Introduction and Methods sections were screened according to the inclusion and exclusion criteria relating to participants, concept, and context. In the fourth stage, the remaining articles were then examined in full against the inclusion criteria. Finally, the reference lists of any articles included at this stage were reviewed, and subjected to the same stages of screening and review until no new articles were identified. Study selection was primarily performed by one author; however, this was conducted under close supervision of the entire research team, who met weekly to review screening decisions. An information specialist/clinical librarian with expertise in scoping review methodologies was also consulted regularly. Moreover, the reviewer adopted a cautious approach, whereby any articles in which there were uncertainties related to meeting inclusion criteria were reviewed by the entire team, and final decisions were made by consensus. Articles included at both stages four and five were screened by three reviewers.

**Table 1:** Inclusion / Exclusion criteria for scoping review papers

	Inclusion Criteria	Exclusion Criteria
*Participants*	Included children aged 0–18 years	Included individuals over the age of 19 years
	Included children with neurodisability (as defined by Morris et al., 2013)	Included children who were typically developing or who had primarily medical diagnoses (e.g., cancer, diabetes)
	Included family/caregivers, including siblings, extended family, and adoptive families	Included paid caregivers
*Concept*	Described connecting children with neurodisability and their families to supports and services in their community	Described a broad concept that was not *specifically* related to connecting children and families to services (e.g., family-centred practice)
*Context*	Described navigation and navigation-type resources, supports and activities relating to connecting children with neurodisability and their families to *community-based pediatric* services, including hospital-to-home/community-based services and across community-based agencies	Described navigation and navigation-type resources, supports and activities relating to connecting children with neurodisability and their families to only *within-hospital* pediatric services or to adult services (e.g., housing, employment)
*Types of Sources*	Peer-reviewed Published after 1990 Published in English Any literature type (empirical, descriptive, literature review, protocol)	Unpublished or grey literature Published before 1990 Published in language other than English

## Data extraction

Data, as specified in the Data Extraction Instrument, were extracted verbatim from included articles into an excel document (see Table 2). In cases in which more than one term was used and described in an article (for example, Ogourtsova et al. [35] included the terms “health coaching”, “service or care coordinator”, “navigator”, and “keyworker”), separate entries were made for each term, with distinct descriptions extracted, if provided by the article authors. This phase of data extraction was piloted in three phases until consensus regarding how to extract data was reached. For each phase, three members of the research team independently extracted data from the same two articles. The team then came together to compare, and through this process, refined the data extraction instrument, and agreed about what constituted the most pertinent data to review. After three rounds (i.e., six articles), consensus was reached. For the remaining 27 articles, data were primarily extracted by a single team member, though group discussion was relied upon when there were uncertainties. Moreover, the larger team collaboratively revisited the data extracted for an additional eight articles to ensure it aligned with the study objectives.

**Table 2:** Data extraction instrument

Title	
Authors/year	
Geographical context of study (i.e., the region where the intervention took place if the article was empirical, or to the authors’ region, if the article was descriptive)	
Literature type	
Study method	
Neurodisability population	
Objective(s)	
Terminology used	
Description(s) provided (e.g., purpose/overarching aims/principles described)	

## Data analysis

In order to address the first part of our review question pertaining to the *terminology* used, the research team utilized a concept-sorting technique for textual data known as cutting and pile sorting [36] as described in previous work [37, 38]. Within this approach, expressions, or in our case extracted terms (e.g., “care coordination”) are pasted onto individual cards and those terms that seem conceptually similar are placed together in piles. This was done virtually using an online card sorting program, Proven by Users (see https://provenbyusers.com). Three members of the research team individually logged in with a unique identifier and independently sorted 49 individual cards, each identified with a distinct term. In order to assist with this stage of sorting, the associated extracted descriptions were also viewable. The compiled results were then reviewed by the research team, and any discrepancies were resolved through discussion until consensus was reached, resulting in a final set of conceptually- and thematically-related terminology groupings.

The second part of our review question focused on the *descriptions* pertaining to navigation-type resources, supports, and activities aimed at connecting children with ND and their families to community-based supports and services. In order to carry out this analysis, data from the ‘Descriptions provided’ category of our data extraction instrument were imported into and analyzed using NVivo 12 for Windows software. Textual data were grouped into related concepts and overarching domains using a constant comparative method [39, 40]. In order to ensure the trustworthiness of the coding method, the domain structure and codebook, which included descriptions and sample quotes, were shared amongst the team throughout the coding process, and coding was continually refined based on weekly team discussions. Moreover, an independent co-coder randomly selected and analyzed 20% of the data, and agreement was found to be acceptable (> 90%) [41–43].

## Results

### Search strategy results

**Fig. 1:**
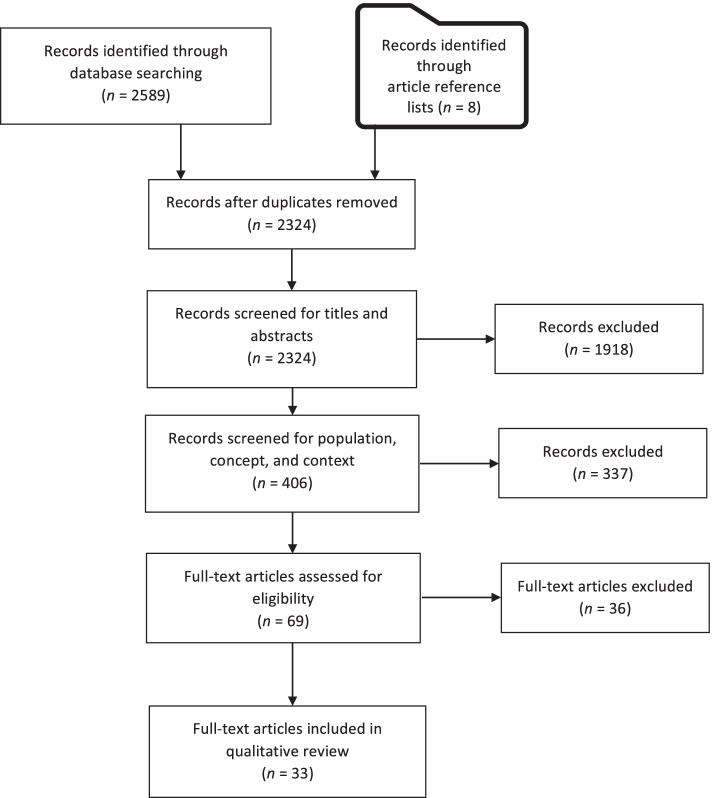
Preferred Reporting Items for Systematic Reviews and Meta-Analysis (PRISMA) flow diagram

The search yielded 2,597 papers, of which 33 met our outlined inclusion criteria with respect to participants, concept, context, and types of sources. Fig. 1 presents the Preferred Reporting Items for Systematic Reviews and Meta-Analysis (PRISMA) flow diagram [44].

### Numbers, sources, and types of papers

Approximately half (*n* = 17; 51%) of the 33 included papers were published since 2010, and most (*n* = 25; 76%) originated from research teams in the United States. A range of literature types was represented, with most (*n* = 21; 64%) presenting empirical data, followed by descriptive papers, commentaries, or editorials (*n* = 9; 27%). Of the 21 articles that presented original research, most (*n* = 13; 62%) employed quantitative methodologies, followed by mixed methods approaches (*n* = 7; 33%). As our inclusion criteria with respect to participant group only specified that children have ND, and the Morris et al. [3] definition we adhered to was quite broad, it was not surprising that the disability populations represented within the included papers were classified and described in various ways. Most frequently (*n* = 17; 52%), the focus of the included articles was on specific diagnosed conditions, with nine papers (27%) targeted to autism spectrum disorder (ASD). Often, however, article authors used nonspecific ways of identifying their participants or populations of interest (*n* = 16; 48%), describing them as children with “developmental disability” (e.g., [45]), “developmental delay” (e.g., [22]), “disabilities” (e.g., [46]), or “special care health needs” (e.g., [47]). Table 3 provides further detail with regard to the yield of papers included.

**Table 3:** Yield of papers by geographical context, literature type, and neurodisability population (*n* = 33)

	References
Geographical Context	
United States (*n* = 25)	[21–23, 45, 47–54, 56, 58–65, 67, 68, 72, 73]
United Kingdom (*n* = 5)	[57, 66, 69–71]
Canada (*n* = 2)	[35, 46]
Australia (*n* = 1)	[55]
Literature Type	
Quantitative (*n* = 13)	[21–23, 45, 50, 52–54, 59, 60, 62, 63, 69]
Descriptive/Commentary/Editorial (*n* = 9)	[35, 46, 47, 49, 56, 64, 65, 72, 73]
Mixed Methods (*n* = 7)	[55, 57, 58, 66, 68, 70, 71]
Literature Review (*n* = 2)	[48, 61]
Qualitative (*n* = 1)	[51]
Study Protocol (*n* = 1)	[67]
Neurodisability Population	
Nonspecific (*n* = 16)	[22, 35, 45–47, 50–53, 58, 62, 64, 65, 69–71]
Autism Spectrum Disorder (*n* = 9)	[21, 23, 48, 49, 54, 59, 67, 68, 72]
Attention Deficit Hyperactivity Disorder (*n* = 3)	[57, 60, 63]
Acquired Brain Injury (*n* = 2)	[55, 61]
Multiple Conditions (*n* = 2)	[56, 66]
Fetal Alcohol Spectrum Disorder (*n* = 1)	[73]

### What terminology is used in the literature?

From 33 included papers, a total of 20 unique terms were extracted that had been used in discourse pertaining to navigation-type resources, supports, and activities aimed at connecting children with ND and their families to community-based supports and services. In order to facilitate more detailed conceptual analysis of potential overlaps and inter-relationships amongst the various terms, extracted terminology were organized into five thematically-related groupings. Only one article, a commentary by Ogourtsova and colleagues [35], included terms that fell within multiple categories, as their aim was to highlight subtleties associated with the commonly used terms: health coaching, keyworker, navigator, and service or care coordinator (see Table 4).

**Table 4:** Unique terminology extracted and associated conceptual groupings

*Conceptual Groupings* and Unique Terms	References
*Coordination* (*n* = 23)	
Care Coordination / Coordinator / Coordinated Care (*n* = 9)	[46, 48–55]
Case Management / Manager (*n* = 9)	[45, 47, 55, 60–65]
Service Coordination / Coordinator (*n* = 4)	[46, 52, 58, 59]
Service or Care Coordinator (*n* = 1)	[35]
Service Integration (*n* = 1)	[46]
Case Planning (*n* = 1)	[55]
Multi-Agency Approach (*n* = 1)	[66]
Patient-Centred Health Care Home (*n* = 1)	[56]
ADHD One Stop Shop (*n* = 1)	[57]
Relational Coordination (*n* = 1)	[59]
Support Broker (*n* = 1)	[59]
*Navigation* (*n* = 6)	
Patient Navigation / Navigator (*n* = 4)	[21–23, 67]
Family Navigation (*n* = 1)	[21]
Navigator (*n* = 1)	[35]
Parent-to-Parent Mentor (*n* = 1)	[68]
*Key Working* (*n* = 4)	
Key Worker / Working (*n* = 4)	[35, 69–71]
*Coaching* (*n* = 2)	
Coaching in Context (*n* = 1)	[72]
Health Coaching (*n* = 1)	[35]
*Family Support* (*n* = 1)	
Family Empowerment (*n* = 1)	[73]
Family Support (*n* = 1)	[73]

*Note.* Reported *n*’s refer to the number of articles

#### Coordination

The largest, and most diverse grouping was comprised of 23 articles using terminology that primarily focused on coordination-related activities. In total, 11 distinct terms were extracted. Nine papers used the term “care coordination/coordinator” or “coordinated care” to indicate the means through which service providers connected families with community resources [46, 48–55]. Others referenced specific models with unique names whose primary focus was on care coordination, and were thus grouped here. For example, Fueyo et al. [56] used the term, “patient-centred health care home (health home)”, which is a proposed model of “lifelong care coordination” through which families of children with ASD and intellectual disability access community resources (p. 1136). Similarly, Sfar-Gandoura et al. [57] evaluated a multi-agency drop-in clinic serving young people with attention deficit hyperactivity disorder (ADHD), entitled “ADHD One Stop Shop”, in which Nurse Specialists were noted to be acting as care coordinators, linking clients with multidisciplinary teams.

Five articles used the term “service coordination/coordinator” [35, 46, 52, 58, 59], with most doing so interchangeably with “care coordination” [46, 52]. For example, Warfield et al. [59] note that depending on the sector within which it is used, “service coordination has also been variously called care coordination, service integration, and case management” (p. 1093). Similarly, Ogourtsova et al. [35] identified this particular branch of service delivery as done by a “service or care coordinator”. King and Meyer [46] differentiated “service integration” from “service coordination”, suggesting that the former refers to the macro-level of service delivery, namely those functions and activities that bring services together, such that they are comprehensive, accessible, available, and unified. In other words, this sets in place an integrated service *system* that individuals and families can access through the clinical process of service coordination. According to these authors, “service integration” occurs at the level of the system, sector, or agency (i.e., macro level); whereas, “service coordination” refers to the process for an individual client and/or family (i.e., micro level). Finally, Warfield et al. [59] used the term, “relational coordination”, identifying it as a measure of “service coordination”, particularly the degree of collaboration and partnership (i.e., shared knowledge and goals, and mutual respect) among service providers. These authors also used the term, “Support Broker” within the Massachusetts home and community-based waiver program, which is a participant-directed program that supports in-home service access for low-income families of young children with severe ASD. The decision was made to place the term “Support Broker” within the ‘Coordination’ grouping, as the overall aim of the study was to evaluate the impact of “coordination efforts” on parent stress and family functioning [59] (p. 1093).

An additional subset of papers was identified (*n* = 9), all of which used the term “case management/manager” [55, 60–64], with some qualifying the specific form as either “nurse case management” [47, 64] or “family-centred case management” [45, 65]. The decision to sort these articles within the ‘Coordination’ grouping followed the observation that almost all explicitly identified that a key responsibility or component of case management was service coordination [47, 55, 60, 62–65]. Thus, the two sets of terms appeared inextricable. For example, in Scheinberg et al.’s [55] survey of pediatric case managers for children with acquired brain injury in Australia, “co-ordination of services” was ranked as the most important component of their case management service. The authors also note that “case management”, “care co-ordination”, and “case planning” have been used interchangeably in the literature. Interestingly, the articles using “case management” were significantly older in comparison to those that expatiated other terminologies, as most (*n* = 5; 56%) papers were published in the early 1990s, and this was in fact the only term extracted from articles published before the year 2000. This area of literature also devoted particular attention to nurses, as five of the papers either referred to nurse case management models [47, 60, 63, 64] or highlighted how nurses could contribute to this role [62].

The final unique term was “multi-agency approach” used by Rowlandson and Smith [66] to refer to a particular project on the Isle of Wight (United Kingdom) initially for children with ASD whose families had experienced a fragmented system of assessment, intervention, and support. This term was placed under the broad ‘Coordination’ grouping as their model employed someone in a “coordinator” position, and primarily focused on coordination in various capacities, including of referrals for assessment and diagnosis, of planning for support and intervention, and of services across agencies.

#### Navigation

Interestingly, although the primary focus of our review was on ‘navigation’ and related activities, only six of the 33 included articles used this particular terminology. In total, four unique terms were extracted under this grouping. One paper indicated that a “navigator” was someone who works with patients and families [35]. Similarly, Feinberg et al. [21] specified that “family navigation” was an adapted form of “patient navigation”; whereas, others evaluated the effectiveness of “patient navigation/navigator” interventions in relation to specified outcomes, including improved service access for children with ASD [23, 67] and increased referrals to early intervention for young children with developmental delay [22]. Moody et al.’s [68] term “parent-to-parent mentor” was grouped within ‘Navigation’, as a central aspect of the study’s intervention was the provision of Navigation Training for parents of children with ASD by “an expert in navigating service systems” [68] (p. 427).

#### Key working

The ‘Key Working’ grouping comprised a relatively small number of papers, but was the most homogenous in terms of utilized terminology, as all four articles used the term “key worker/working” [35, 69–71]. With the exception of Ogourtsova et al. [35], whose paper provided a descriptive review of various terms, the other papers were empirical. Beecham et al. [69] estimated the unit costs for key worker services for children with various neurodevelopmental diagnoses in the United Kingdom (UK), Rahi et al. [70] reported on the impact of a hospital-based key worker service on parents’ experiences and health care professionals’ practices, and Young et al. [71] reviewed findings from an evaluation of a key working model in the UK entitled Early Support.

#### Coaching

 The ‘Coaching’ grouping was comprised of two unique terms that were extracted from two articles. Ogoutsova et al. [35] reviewed definitions of “health coaching” for children with developmental disabilities and their families, identifying this as a branch of health-care service that is distinct from service/care coordination, navigation, and key working. Potvin et al.’s [72] “coaching in context” model incorporated coaching and context therapy for children with ASD and their families.

#### Family support

For ‘Family Support’, two inter-related, yet distinct, terms, “family support” and “family empowerment”, were extracted from the same article, which referred to a specific information, referral, and support network program for children with fetal alcohol spectrum disorder and their families [73]. As “family empowerment” was explicitly specified by the article authors as a component of “family support”, these terms were placed together under the same conceptual terminology grouping.

### What descriptions have been provided?

This component of the analysis was focused on the descriptions provided in the literature pertaining to navigation and related terms. In particular, we reviewed descriptions of the purpose, overarching aims, and principles that were included within the reviewed articles to accompany the various terms identified above (see Table 2). Across the five terminology groupings, four central domains emerged, namely: (*i*) what navigation-related resources, supports and services aim to *facilitate* and *(ii) provide; (iii)* descriptions of their *intended outcomes;* as well as (iv) descriptions of their *guiding principles* (see Table 5).

**Table 5:** Domains describing navigation and related work within childhood neurodisability

Facilitate:
Integration / coordination of resources, supports, and services within and across disparate and complex services, agencies, and systems Identification of individualized needs Identification and reduction of barriers to access
Provide:
Information, advice, and education Single point of contact Emotional support Advocacy
Intended Outcomes:
Improved health, behaviour, and capacity Decreased patient and family distress Increased satisfaction with services
Guiding Principles:
Client-directed, family-centred, and collaborative May be brief and time-limited or longitudinal

#### Facilitate

The most frequently discussed domain fell within what we deemed, ‘Facilitate’, and was coded within 29 of the 33 included articles (89%). Article authors most frequently described their role in working to facilitate integration and coordination of resources, supports and services within and across disparate and complex services, agencies, and systems. This domain was referred to within 26 articles (79%), with authors describing how navigation and related activities sought to bridge gaps along an individual and family’s care or service pathway, promote continuity of service engagement, reduce disparities in access, and improve the efficiency with which limited resources could be accessed. This work occurred within and across various agency lines, sectors, and systems, and involved understanding complex logistics around service mandates and scope. The next most frequently described sub-domain was observed within 12 articles (36%), and centred on how navigators and related professionals facilitated the identification of the child and family’s individualized needs and preferences, so services could be tailored appropriately. Finally, six articles (18%) included descriptions referencing the role of assisting ‘clients’ (i.e., individuals and families) to overcome or eliminate patient-specific barriers to care (“facilitate identification and reduction of barriers to access”). McAllister et al.’s [51] description of “care coordination” includes all three sub-domains, as they indicate that within their program, “a multidisciplinary team worked with families of children with neurodevelopmental disabilities to collaborate across tertiary and community organizational boundaries and barriers to effectively marshal available resources”, and was designed “to facilitate access to needed interventions and services, eliminate barriers to care, address unmet needs, and clarify shared responsibilities for a population with complex needs” (p. 89).

Further, we examined which domains were particularly prominent for each of the five terminology groupings identified and discussed above (i.e., domains that were coded within approximately half of the included articles for a particular terminology grouping). Descriptions that referred to facilitating integration and coordination of resources, supports, and services within and across disparate and complex services, agencies, and systems were prominent for all terminology groupings, with the exception of ‘Family Support’ (for more information about the distribution of domains across terminology groupings, see Supplementary Table). Descriptions regarding identifying individualized needs were prominent within both ‘Coaching’ and ‘Coordination’, and finally, only articles pertaining to ‘Navigation’ referred to the identification and reduction of barriers to access.

#### Provide

Term descriptions also reflected the specific kinds of supports ‘Provided’ by navigators, with this domain being represented within 15 articles (45%). Ten articles (30%) contained descriptions that focused on the provision of education, advice, and information tailored to individual and family needs, such as on behaviour management or approaches to child discipline [64]. Across terminology groupings, this domain was most prominent for articles describing ‘Coaching’, ‘Key Working’, and ‘Navigation’. Eight articles (24%) explicitly mentioned that the professional served as a “single point of contact” for families, thus reducing their need to seek multiple referrals to various services across agencies, and this was most prominent for ‘Coaching’ and ‘Key Working’. Seven of the included articles (21%) described the provision of emotional support, with Steele [47] saying that the “nurse case manager also acted as a counselor and confidant to the families” (p. 615). Discussions of emotional support were most prominent within articles pertaining to the ‘Key Working’ and ‘Navigation’ terminology groupings. Finally, three articles (9%) mentioned the role of advocacy, including two related to ‘Coordination’ and one pertaining to ‘Key Working’. These papers described the necessity of assuming the ‘advocate’ role on behalf of families at times.

#### Intended outcomes

The ‘Intended Outcomes’ of these navigation-related practices was also a frequently identified domain, being present within 21 of the included articles (64%). Within this domain, descriptions of the overall goal of improving child and family well-being, including improved physical and emotional health, child behaviour, and importantly, family capacity emerged as important, and were present within 17 articles (52%). This particular domain was prominent within each terminology grouping, with the exception of ‘Navigation’. Accompanying descriptions often noted the importance of assisting parents with acquiring skills that would empower and foster their independence, thus improving their capacity for self-care and confidence to advocate for themselves. For example, Fiene et al. [65] wrote, “The goal of case management is client empowerment – that is, teaching needed skills so that clients and families develop the self-efficacy that enables them to be in control of their own service and habilitation program” (p. 324). Seven articles (21%) described the goal of decreasing patient distress, including parent stress, anxiety, and sense of burden. Finally, three articles (9%), including two related to ‘Coordination’ and one pertaining to ‘Navigation’, explicitly mentioned the intention to improve patient satisfaction with services, such that families would perceive them as more integrated, effective, and efficient. For example, King & Meyer [46] wrote that “Service integration and service co-ordination share an ultimate common goal – to enhance the likelihood that clients will perceive care to be easy to access, seamless, and tailored to their needs” (p. 479).

#### Guiding principles

Finally, approximately half (*n* = 18; 55%) of the included articles’ descriptions reflected on the ‘Guiding Principles’, with most (*n* = 15; 45%) indicating that it should aim to be client-directed, culturally responsive, and family-centered. The descriptions focused on the centrality of the client and family, and the importance of collaboration and partnership. Service providers had to work to develop trust and maintain open communication with the families they partnered with, in order for families to be comfortable sharing their beliefs and concerns. This domain was most prominent within descriptions accompanying the terminology groupings of ‘Family Support’, ‘Coaching’, and ‘Coordination’, and is exemplified by Potvin et al. [72] who wrote, “Family-driven practice involves families having the primary decision making role in all aspects of care including setting goals and designing, implementing, and evaluating their child’s intervention plan. Culturally responsive practice includes understanding a person’s beliefs for the medical condition they experience, recognizing their cultural identity, developing a trusting relationship, and using strengths-based approaches” (p. 48). Six articles (18%) also included specific indications of the temporal nature of their work as either brief and time-limited (*n* = 4) or longitudinal in nature (*n* = 3). Interestingly, 3 of the 4 articles using the former description referred to ‘Navigation’ [21, 35, 67], and all that described their work with families as a long-term partnership accompanied terms falling within the ‘Coordination’ grouping [35, 56, 64].

## Discussion

Children with ND often have significant service requirements, and their families are challenged with managing and coordinating supports acquired from various sources and sectors, a role that has been identified as burdensome and overwhelming. As such, there is a need for some kind of agent or agency to support, partner and guide families to the extent desired in their quest to obtain appropriate information and services. This role may best fall under the scope of patient navigation (PN), as the aims of PN approaches align with such needs, and the few studies empirically evaluating their efficacy for those with ND suggest they are well suited [20–23]. Our understanding of the nature and effectiveness of these interventions, however, is marred by the diversity of terminology used to describe similarly-intended approaches. The current scoping review aimed to examine the terminology and descriptions available in the literature relating to navigation and ‘navigation-type’ resources, supports, and activities aiming to connect children with ND and their families to community-based supports and services. We suggest that this is an important first step in clarifying areas of potential distinction and overlap among the various related branches of practice, with the hope that this can inform how such services are developed, implemented, and evaluated for families of children with ND.

### Terminology

Across the articles that met our inclusion criteria, a varied range of terminology was used to refer to the practice of connecting children and families to community services, though the individual terms could be grouped under broad conceptually related categories that are familiar from the literature [28, 35], as well as from community-based practical settings. Although the initial focus of the review was on ‘navigation’, a preliminary search indicated that articles in the field used a variety of keywords and subject headings, and thus it seemed pertinent to include these other, related terms in our search queries. Indeed, only a small proportion of relatively recent publications actually utilized the term “navigation”. It is interesting that although the field of navigation emerged in the early 1990s with Dr. Freeman’s work, it does not appear to have been applied to the ND population context, at least empirically, until decades later. In the current review, the oldest navigation paper included was published very recently – in 2016. This is consistent with Gardiner & Miller [18], who noted that children with ND are not as well represented within this body of literature, despite their experiences with fragmented systems and documented need for coordination.

Although many distinct terms were used within the reviewed literature, there was great overlap in how terms were used, and at times this was done interchangeably so within the same article. This was particularly apparent when examining papers related to care coordination, service coordination, case management, and case planning. Interestingly, the included “case management” literature was often older and appeared particularly tied to the field of nursing. According to Cesta [74], the concept of case management has been in existence for almost a century, originating in community-based psychiatric, social work, and public health nursing settings, but by the 1990s evolved to most often be implemented within acute care by nursing staff. In relation to childhood ND, case management first became central when Public Law 99–457 (PL 99–457), the Education for the Handicapped Act, was passed in 1986 in the United States, which required that a case manager be named as part of the Individualized Family Service Plan [64, 75]. The earlier publications included in this review, particularly by Steele and colleagues [47, 62, 64], were thus at the precipice of describing and evaluating what was at the time, a relatively new approach to meeting the needs of those with ND and their families. It was clear that service or care coordination was a central aspect of case management, and this is consistent with the Case Management Society of America’s (CMSA) definition, which states:

Case management is a collaborative process of assessment, planning, facilitation, care coordination, evaluation, and advocacy for options and services to meet an individual’s and family’s comprehensive health needs through communication and available resources to promote quality cost effective outcomes. [76]

In contrast to the diversity of terminology represented within the ‘Coordination’, and to some extent, ‘Navigation’ groupings, terms relating to ‘Key Working’, ‘Coaching’ and ‘Family Support’ were relatively homogeneous. These terminology groupings were represented in just a few articles, which most frequently described or evaluated specific program models. Because coaching models including families of children with ND have most often focused on parenting interventions or parent-mediated interventions intended to improve child functioning and behaviour [35, 72], it is not surprising that relatively few articles described this model in relation to connecting to community-based services. It was interesting and somewhat surprising that only one article using terminology related to ‘Family Support’ was included in the final review. We suspect that this is most related to our strict inclusion criteria that in order to be included, a paper had to describe the practice of connecting children and families to supports, services, or resources in their communities. ‘Family Support’ may best be conceptualized as a higher-level umbrella term under which the various kinds of navigation, care coordination, coaching, and key working supports are situated.

## Descriptions

When examining the descriptions that accompanied the varied terms, it was immediately apparent that even when the same term is used, it was described in rich and diverse ways. However, there were core domains that emerged within the descriptions article authors provided, and these painted a detailed picture of the core aspects underscoring this work. Authors described how professionals working in these related fields aimed to facilitate integration and coordination of complex and disparate services, as well as identify individualized needs and reduce family-specific barriers. This work was guided by clear goals, with far-reaching intended outcomes that included improved health, child behavior, and family capacity, decreased distress, and increased satisfaction with services. Guiding principles also emerged, including being client-directed, family-centred and collaborative, and professionals operating under the ‘navigation and related services’ umbrella provided various supports and services, including information, advice, education, emotional support, and advocacy, and supported families by being their single point of contact. We propose this four-domain ‘model’ (see Table 5) as a useful, empirically-informed conceptualization of this role, which has been lacking from the literature, particularly as related to the childhood ND context.

Gardiner and Miller [18] provided an outline of the core roles and attributes of the navigator role as applied within childhood ND; however, this was based on an overview of the literature, and the expansion provided in this paper provides a much-needed and importantly, *empirically-based*, update. Specifically, they suggested that navigators facilitated access to resources, but did not include the need for services to be integrated and coordinated. This is clearly a critical component of this work, as it was identified in the majority of the included articles, and we suggest better reflects one of the core challenges families have – that services are siloed, as well as the reality that needed services for children with ND must be accessed from multiple sectors and agencies. Gardiner and Miller also did not mention the values guiding this work, including being client-directed, family-centred and collaborative, or the intended outcomes, both of which were clearly important and prominent elements within article descriptions. Moreover, inclusion of these outcomes related to improved well-being and capacity are consistent with Rollins et al. [19], whose detailed conceptual impact model outlines the multifactorial ways in which PN can address commonly encountered barriers facing this population, and the anticipated benefits for children and their families.

As described, there was significant overlap in the descriptions provided across the five conceptually-related terminology groupings, with little to identify one grouping as distinct from the others. However, when we examined what domains were particularly prominent for each, some nuanced distinctions were apparent (see Table 6). For example, ‘Key Working’ was distinguished by the fact that all papers noted that key workers act as the *single point of contact* for families. ‘Coaching’ was most notably focused on *building family skills* with the goal of improving behavior, and being client-directed, family-centred, and collaborative in approach. ‘Navigation’ is a *time-limited* service that aims to *reduce barriers* preventing families from successfully accessing services, and provides information, advice, education, and emotional support. Finally, ‘Coordination’ was the most *multidimensional*, and yet the only domain distinguishing it from the others was its *longitudinal* nature. ‘Coordination’ may be the most comprehensive approach to connecting families to service, as prominent domains included integration of services across disparate systems and agencies, a focus on improved health and behavior, and acting in client-directed, family-centred, and collaborative ways.

**Table 6:** Domains distinguishing terminology groupings

Terminology Grouping	Distinguishing Domains
Key Working	•Key workers act as the single point of contact for families •Key workers are focused on building family capacity
Coaching	•Most notably focused on building family skills with the goal of improving behavior •Coaches are client-directed, family-centred, and collaborative in approach
Navigation	•This is usually a time-limited service •Navigators aim to reduce barriers preventing families from successfully accessing services •Navigators provide information, advice, education, and emotional support
Coordination	•Coordination emerged as the most multidimensional and comprehensive set of services •Coordination was distinguished by its longitudinal approach •Prominent domains included integration of services across disparate systems and agencies, a focus on improved health and behavior, and acting in client-directed, family-centred, and collaborative ways

*Note.* As only one article used the term “Family Support”, we were unable to analyze what distinguished it from other terminology groupings

### Proposing an integrative model of navigation and related practices

**Fig. 2:**
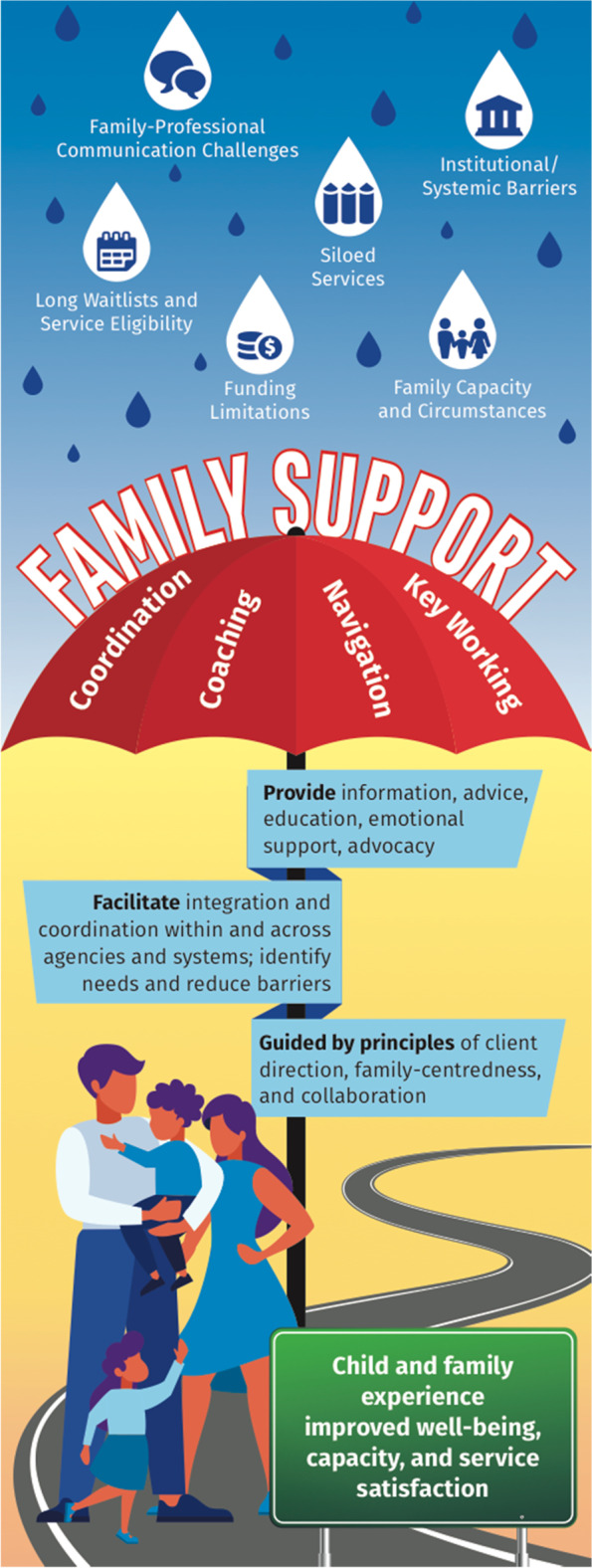
Integrative Model of Navigation and Related Practices

In conclusion, we propose an integrative visual model that synthesizes the findings from this scoping review (see Fig. 2). Various iterations of this visual have been presented to the stakeholders originally consulted at the start of this project, as well to other, broader stakeholder groups, including to family members of children with ND, service providers, government personnel, and academic researchers. Fig. 2 showcases ‘Family Support’ as the overarching umbrella under which the specific, but related practices of ‘Coordination, ‘Coaching’, ‘Navigation’, and ‘Key Working’ reside. This umbrella serves to support families to face various barriers in their quest to procure needed services for their children with ND. The umbrella post is flagged with the central domains underscoring these family support-related practices, identified in the literature, including providing information, advice, education, emotional support, and advocacy; facilitating integration and coordination within and across agencies and systems, identifying needs, and reducing barriers; and the principles guiding the work, including being client-directed, family-centred, and collaborative. In an ideal world in which these practices can be executed without systemic barriers such as service provider caseloads, funding restrictions, and cross-agency communication limitations, the family is shielded from the barriers they face, and are supported to experience the ‘intended outcomes’ described within the reviewed papers, including improved health and well-being, capacity, and increased satisfaction with services.

We envision this model as a dynamic one, which frames the ‘lay of the land’ of PN and related approaches specifically pertaining to children with ND and their families. In practice, this framework, in concert with the details shared in Table 6, may assist professionals in their communications with families about the range of supports they can offer, as well as the family-centred goals they can collaboratively work toward. In research, our intention is that this framework distils the essential points from the limited literature pertaining to navigation for this population, but that also reflects the broader literature associated with related terminologies. We hope that this framework may be used to inform program development and evaluation research, and can continue to evolve as guided by the expertise and lived experiences of professionals and families.

### Limitations and future directions

We acknowledge potential limitations associated with the current scoping review. The first limitation relates to the employed search strategy, which included varied terminology (as opposed to limiting it to the term “navigation”) and was conducted in June 2019. Although a vast variety of terms emerged, it is possible that additional related terms were missed and that newer relevant literature was not captured within our scoping review. Future research that can periodically review and update our proposed navigation framework such that it reflects the latest research and breadth of terminology will help to continually advance the field.

Second, although done in close consultation and collaboration with the larger research team, a single reviewer completed much of the article screening and data extraction. Articles, however, were collaboratively reviewed and checked to ensure that they met our inclusion criteria and that data had been extracted reliably, and the team met frequently to review progress and resolve any uncertainties.

A final limitation may be related to the exclusion of grey literature. This decision was made as this research group is conducting a concurrent environment scan, examining the descriptions of navigation and related activities, as implemented within programs and agencies in a Western Canadian province. Future research that includes and reviews relevant unpublished, non-peer-reviewed literature could be very helpful in informing the field as to how these terms are used and described *in practice*. Another direction that may help to advance our understanding of practice would be an analysis of the service ‘types’ offered across various terminologies. As this review included both empirical and descriptive papers, we did not analyze the specific types of services delivered under each terminology umbrella, as this information was not always present. Future research that can explore how services differ, or not, according to their title, will strengthen our understanding of the potential nuances differentiating ‘navigation’ from ‘coordination’, ‘family support’, ‘coaching’, and ‘key working.’

## Conclusions

The current paper reviewed terminology and descriptions present in the literature pertaining to the ways in which agents or agencies may assist children with ND and their families to connect to community-based supports and services. The observed data provided insight into how these terms have been used, informing our understanding of the core domains characterizing the work broadly, and highlighted areas of distinction and overlap. Importantly, the observation of such variability in relation to pertinent ‘navigation’ and ‘navigation-related’ terminology and their associated descriptions highlights the importance of harmonizing the efforts of multiple and diverse stakeholders, in order to make progress for children with ND and their families. Within this review we also proposed an integrative model synthesizing the diverse terminology and descriptions characterizing the field of ‘navigation’ and related practices serving children with ND and their families. We hope this framework can be used to guide mindful characterization of how supports aiming to connect children and families to needed service are termed and described in future research and in practice. The review provides an empirically-based first step to characterizing these branches of service, and addresses a significant gap in the literature with regard to childhood ND.

## Supplementary Information

**Additional file 1. **

## Data Availability

The datasets used and/or analysed during the current study are available from the corresponding author on reasonable request.

## Abbreviations

ADHD: Attention Deficit Hyperactivity Disorder
ASD: Autism Spectrum Disorder
CMSA: Case Management Society of America
ND: Neurodisability
PN: Patient Navigation
PRISMA: Preferred Reporting Items for Systematic Reviews and Meta-Analysis
UK: United Kingdom

## Footnotes

1 Prevalence estimates for children with ND vary from 4.5% [1] to 1 in 6 children [2], depending on definition used and source of data.
