# Supplementary material for: Terminology and descriptions of navigation and related practices for children with neurodisability and their families: a scoping review
Source: BMC Health Serv Res. 2022 Feb 17;22:214. doi: 10.1186/s12913-022-07617-y (PMC8851781; doi:10.1186/s12913-022-07617-y)
Supplement: Supplementary file 1 — Additional file 1. [file 12913_2022_7617_MOESM1_ESM.docx]

**Supplementary Table**

Distribution of Description Domains across Terminology Groupings

|  | **Coordination**  **(*n* = 23)** | **Navigation**  **(*n* = 6)** | **Key Working**  **(*n* = 4)** | **Coaching**  **(*n* = 2)** | **Family Support**  **(*n* = 1)** |
| --- | --- | --- | --- | --- | --- |
| *Facilitate:* |  |  |  |  |  |
| Integration / coordination of resources, supports, services within and across complex services, agencies, and systems | 19 | 4 | 4 | 1 | - |
| Identification of individualized needs | 10 | - | 1 | 1 | - |
| Identification and reduction of barriers to access | 2 | 4 | 1 | - | - |
|  |  |  |  |  |  |
| *Provide:* |  |  |  |  |  |
| Information, advice and education | 5 | 3 | 2 | 1 | - |
| Single point of contact | 4 | - | 4 | 1 | - |
| Emotional support | 2 | 3 | 2 | - | - |
| Advocacy | 2 | - | 1 | - | - |
|  |  |  |  |  |  |
| *Intended outcomes:* |  |  |  |  |  |
| Improved health, behaviour, and capacity | 12 | 1 | 2 | 2 | 1 |
| Decreased patient and family distress | 5 | 1 | 1 | - | - |
| Increased satisfaction with services | 2 | 1 | - | - | - |
|  |  |  |  |  |  |
| *Guiding Principles:* |  |  |  |  |  |
| Client-directed, family-centred, and collaborative | 10 | 2 | 1 | 2 | 1 |
| Brief and time-limited | 1 | 3 | - | - | - |
| Longitudinal | 3 | - | - | - | - |
